# Harnessing CAR-Extracellular Vesicles for Next-Generation Cancer Immunotherapy

**DOI:** 10.3390/ijms27052163

**Published:** 2026-02-25

**Authors:** Sharenya Chelvaretnam, Kol Thida Mom, Carlos Andres Palma Henriquez, Quang Pham, Amirah Fitri, Sadman Bhuiyan, Mozhgan Shojaee, Kartini Asari, Hiba Rashid, Leearne Hinch, Ramin Khanabdali, Gregory Rice

**Affiliations:** 1 INOVIQ Ltd., Melbourne, VIC 3168, Australia; schelvaretnam@inoviq.com (S.C.); kmom@inoviq.com (K.T.M.);; 2 Centre for Clinical Research, Faculty of Medicine, The University of Queensland, Brisbane, QLD 4006, Australia

**Keywords:** cancer, chimeric antigen receptor, extracellular vesicles, immunotherapy

## Abstract

Cancer immunotherapy has experienced substantial progress in recent years, particularly with the advancement of chimeric antigen receptor (CAR) technology, which enables immune cells to selectively target tumor-associated antigens. CARs, now in their fifth generation, are engineered by combining monoclonal antibody fragments with signaling and co-stimulatory domains and have been successfully applied to T cell, natural killer (NK) cell, and macrophage-based therapies. Notable clinical successes, such as tisagenlecleucel and lisocabtagene maraleucel underscore the therapeutic potential of CAR-T, CAR-NK and CAR-macrophages (CAR-Ms), which are currently being evaluated in numerous clinical trials. One promising extension of this approach involves the use of extracellular vesicles (EVs) derived from these immune cells. These nano-sized vesicles offer a cell-free platform to deliver diverse anticancer mediators, addressing the complex and dynamic nature of tumor environments. In this review, we examine the therapeutic potential and immunogenic properties of CAR-derived EVs, along with their role in modulating immune responses. Furthermore, we explore their application as targeted delivery vehicles for chemotherapeutic agents, with the goal of enhancing anti-tumor efficacy while minimizing systemic toxicity.

## 1. Introduction

Cancer immunotherapy has undergone remarkable advancements in recent years, revolutionizing the landscape of cancer treatment. Among the innovative approaches, extracellular vesicles (EVs) derived from genetically engineered chimeric antigen receptor T (CAR-T), natural killer (CAR-NK) cells and CAR-macrophages (CAR-Ms) have emerged as a promising avenue. These cell-derived nanovesicles provide a unique cell-free platform, diversifying the arsenal of anticancer mediators. In this review, we first examine the clinical success and challenges associated with CAR-immune cell therapies in both solid and hematological malignancies. We then focus on the therapeutic utility of CAR-immune cell-derived EVs (CAR-EVs), highlighting how they may overcome several limitations of their parent cells. Furthermore, we explore the potential of CAR-EVs as targeted delivery vehicles for chemotherapeutic agents, aimed at enhancing anti-tumor efficacy while reducing systemic toxicity.

## 2. CAR-T, CAR-NK and CAR-Macrophages: Revolutionizing Cell Therapy

CAR-based immunotherapies, encompassing CAR-T, CAR-NK and CAR-M platforms, have redefined the landscape of adoptive cellular therapy. However, their broader application, especially in the treatment of solid tumors, remains constrained by a constellation of biological, technical, and translational challenges. These include issues related to tumor antigen heterogeneity, immunosuppressive microenvironments, limited in vivo persistence, and complex manufacturing logistics, all of which collectively hinder the scalability and generalizability of CAR-based therapies across diverse oncologic indications. In the following sections, these multifaceted limitations will be examined in detail, with particular emphasis on the mechanistic underpinnings and translational implications for CAR-engineered cell therapies.

### 2.1. Evolution of Chimeric Antigen Receptors

The essential components of CARs include an extracellular antigen recognition domain, typically derived from a single chain fragment variable (scFv) of a monoclonal antibody; a transmembrane domain such as CD3ζ, CD4, CD8α, or CD28; and an intracellular signaling domain responsible for T cell activation [1].

**Figure 1:**
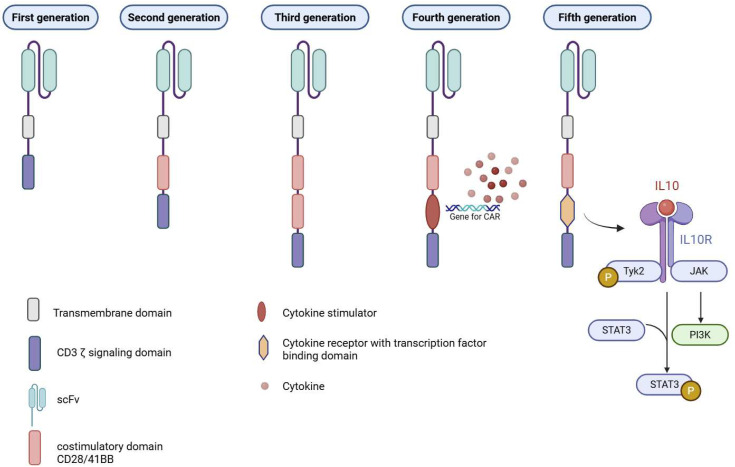
An overview of the different generations of CARs, each representing structural and functional advancements. Schematic showing five generations of CARs. First-generation CARs include CD3ζ alone; second and third generations contain one or two co-stimulatory domains (e.g., CD28, 41BB). Fourth-generation CARs (TRUCKs) express inducible cytokines that are secreted upon antigen recognition to boost local anti-tumor immune responses. Fifth-generation CARs activate JAK-STAT signaling (e.g., STAT3 via IL-10R-like signaling) via a modified cytokine receptor domain. Created in BioRender. https://BioRender.com/fdcd8wk (accessed on 20 December 2025).

There are currently five generations of CARs, each representing structural and functional advancements (Figure 1). The first generation consists of an scFv antigen-binding domain linked to a CD3ζ signaling domain. The second generation builds on this by incorporating a single co-stimulatory molecule, such as CD28, CD134 (OX40), or CD137 (4-1BB), to enhance T cell activation and persistence, while the third generation combines multiple co-stimulatory domains to further improve CAR-T cell function. Fourth-generation CARs, also known as TRUCKs (T cells Redirected for Universal Cytokine Killing), include a co-stimulatory domain along with a transcription factor that promotes cytokine production and broader immune activation. Finally, fifth-generation CARs integrate an intracellular domain from cytokine receptors that contains a binding site for transcription factors, thereby activating the JAK-STAT signaling pathway to support T cell proliferation, survival, and functionality [1].

Likewise, functional modifications developed for CAR-T cells are similarly being explored in CAR-NK and CAR-M platforms. However, these modifications are often adapted to leverage the distinct biology of each innate immune cell type. While NK cells have been engineered to enhance cytotoxic persistence and trafficking, CAR-Ms have been engineered to promote phagocytosis, inflammatory polarization, and tumor microenvironment (TME) remodeling [2,3,4,5]. Thus, while the conceptual framework of CAR optimization is shared, the functional objectives and implementation are cell-type specific.

### 2.2. Tumor Microenvironment (TME) and Immune Evasion

The immunosuppressive TME represents a profound and multifactorial barrier to the therapeutic efficacy of CAR-immune cells, primarily by orchestrating a dynamic network of immune evasion strategies. Within this pathophysiological niche, immunoregulatory cellular constituents—including regulatory T cells (Tregs), myeloid-derived suppressor cells (MDSCs), and tumor-associated macrophages (TAMs)—secrete a repertoire of suppressive cytokines such as transforming growth factor-β (TGF-β) and interleukin-10 (IL-10), which collectively subvert the cytotoxic functionality, persistence, and metabolic fitness of adoptively transferred CAR-modified effectors [6,7].

Concomitantly, the aberrant expression of immune checkpoint ligands, notably the programmed death-ligand 1 (PD-L1), on both malignant and stromal compartments, precipitates a state of functional exhaustion in CAR-T cells, characterized by diminished proliferative kinetics and attenuated effector cytokine secretion [8]. Although CAR-NK cells exhibit a relatively reduced susceptibility to classical checkpoint inhibition, their anti-tumor activity is nonetheless constrained by tumor-mediated modulation of ligand expression, specifically the downregulation of activating ligands such as major histocompatibility complex (MHC) class I chain-related protein A (MICA) and MHC class I chain-related protein B (MICB) and the upregulation of inhibitory ligands like human leukocyte antigen E (HLA-E), which engage NK cell inhibitory receptors including NKG2A, thereby dampening cytolytic responses [9].

Moreover, the TME imposes a metabolically adverse landscape, typified by hypoxia, nutrient deprivation, lactic acidosis, and elevated extracellular adenosine, all of which converge to impair mitochondrial bioenergetics, disrupt glycolytic flux, and promote the epigenetic and transcriptional reprogramming of CAR-expressing cells toward dysfunctional or terminally exhausted phenotypes [10,11]. These cumulative immune-metabolic and immunoregulatory constraints necessitate the rational design of next-generation CAR constructs, such as armored CARs, checkpoint-resistant variants, or metabolically reprogrammed effectors, and the integration of combinatorial therapeutic modalities to overcome TME-induced resistance and enhance the durability of CAR-immune cell-mediated tumor eradication [12,13].

### 2.3. Limited Persistence and Expansion

The limited persistence and expansion of CAR-T cells in vivo remains to be another pivotal constraint undermining their long-term therapeutic efficacy, particularly in the context of solid tumors. This phenomenon is multifactorial, encompassing intrinsic cellular exhaustion, suboptimal memory T cell formation, and the absence of sustained antigenic stimulation. Upon infusion, CAR-T cells often undergo rapid contraction following initial expansion, a process exacerbated by terminal differentiation and the upregulation of exhaustion markers such as programmed cell death protein 1 (PD-1), T cell immunoglobulin and mucin-domain containing-3 (TIM-3), and lymphocyte-activation gene 3 (LAG-3) [14]. Moreover, the immunosuppressive milieu of the TME, characterized by high levels of TGF-β and adenosine, further impairs CAR-T cell metabolic fitness and cytokine production, thereby curtailing their proliferative potential [13].

In addition, the manufacturing process itself may predispose CAR-T products to limited persistence. Ex vivo expansion protocols often favor effector over central memory phenotypes, which are less durable in vivo [15]. Strategies to enrich stem cell memory T cells (T SCM) or to incorporate cytokine support (e.g., IL-7 and IL-15) during expansion are under investigation to enhance persistence [16,17]. Furthermore, the use of humanized or fully human CAR constructs, as well as gene editing approaches to disrupt inhibitory pathways (e.g., PD-1 knockout), are being explored to mitigate exhaustion and prolong functional longevity [18]. These limitations underscore the necessity for iterative optimization of CAR-T cell design and manufacturing to achieve durable responses, particularly in the treatment of solid malignancies.

In contrast to CAR-T cells, CAR-NK cells and CAR-Ms exhibit fundamentally different persistence and expansion dynamics in vivo, which shape both their therapeutic potential and safety profile. NK cells are intrinsically short-lived effectors, with physiological persistence measured in days to weeks rather than months [19]. Consequently, CAR-NK cells generally demonstrate limited in vivo expansion and durability, largely due to their dependence on exogenous cytokine support (e.g., IL-2 or IL-15) and their reduced capacity to form long-lived memory populations compared to T cells [20]. While this limited persistence may constrain sustained tumor control, it also reduces the risk of prolonged toxicity, including cytokine release syndrome and on-target off-tumor effects, making CAR-NK cells particularly attractive for allogeneic “off-the-shelf” applications [21].

CAR-Ms, by contrast, are not designed to expand clonally in vivo, and their persistence is governed primarily by tissue residency, survival cues, and local differentiation states rather than proliferation [22]. While this inherently limits their numerical persistence, macrophages possess superior longevity within tissues and are highly adapted to survive within the hostile metabolic and immunosuppressive conditions of the TME. Importantly, CAR-Ms are less prone to exhaustion in the classical sense but can undergo phenotypic reprogramming toward immunosuppressive or pro-tumoral states under sustained TME influence [4,23]. Engineering strategies aimed at reinforcing pro-inflammatory (M1-like) polarization, enhancing resistance to TGF-β signaling, or enabling autocrine cytokine support are being explored to stabilize CAR-M function over time [24,25].

### 2.4. Antigen Escape and Tumor Heterogeneity

Antigen escape and tumoral heterogeneity also constitute major impediments to the sustained efficacy of CAR-T, CAR-NK and CAR-M cell therapies. Tumor cells can evade immune-mediated cytotoxicity by downregulating or entirely abrogating the expression of the targeted antigen, a phenomenon well-documented in both hematologic and solid malignancies. In B cell leukemias and lymphomas, for example, relapse following CD19-directed CAR-T therapy is frequently associated with the emergence of CD19-negative clones, underscoring the selective pressure imposed by mono-specific targeting strategies [26,27]. Similarly, in solid tumors, heterogeneous expression of antigens such as human epidermal growth factor receptor 2 (HER2) or epidermal growth factor receptor (EGFR) can result in partial tumor clearance and the subsequent expansion of antigen-negative variants [28,29].

This challenge is further compounded by the spatial and temporal variability of antigen expression, driven by clonal evolution, epigenetic reprogramming, and microenvironmental cues. Immunophenotyping analyses have revealed that even within a single tumor lesion, distinct subpopulations may exhibit divergent antigenic landscapes, rendering them differentially susceptible to CAR-mediated recognition and killing [30]. Such heterogeneity necessitates the development of more adaptable therapeutic platforms, including multi-specific or switchable CAR constructs, as well as combinatorial approaches that incorporate immune checkpoint inhibitors or epigenetic modulators to pre-empt or overcome antigen escape [31]. Without addressing these adaptive resistance mechanisms, the long-term durability of CAR-based immunotherapies will remain fundamentally constrained.

### 2.5. Manufacturing and Scalability Constraints

The manufacturing and scalability of CAR-T cell therapy are constrained by its inherently autologous nature, necessitating the individualized collection, genetic modification, and expansion of patient-derived T cells. The successful manufacture of functional CAR-T cells hinges on the quality and composition of the autologous T cell starting material, which is often compromised in cancer patients due to age, disease burden, and prior lymphotoxic treatments. These factors can lead to diminished memory T cell populations and unpredictable yields, complicating manufacturing outcomes [32]. Cryopreservation, while logistically advantageous, can reduce cell viability and recovery compared to fresh products, though it may also selectively eliminate suppressive cell types such as neutrophils and myeloid-derived suppressor cells [33]. However, fresh apheresis products pose logistical challenges due to their limited viability window. Additionally, the presence of inhibitory cellular subsets, such as MDSCs, monocytes, granulocytes, red blood cells, and regulatory T cells, can impair T cell activation and expansion ex vivo [34,35]. Critically, inadvertent transduction of malignant cells during manufacturing poses a serious risk, potentially generating CAR-expressing tumor cells that evade immune targeting. Studies have indicated that there is a small but real risk of developing secondary malignancies after CAR-T cell therapy. These are primarily hematological malignancies, such as myelodysplastic syndromes (MDS), which can arise from adverse gene integration events during the CAR-T transduction process [36]. Therefore, rigorous purification of the starting material, including lymphocyte enrichment and elutriation, is essential to ensure the safety, efficacy, and consistency of CAR-T cell products. This bespoke process is not only time-intensive (i.e., often requiring several weeks from leukapheresis to infusion) but also susceptible to logistical bottlenecks and manufacturing failures, particularly in patients with heavily pretreated or lymphogenic profiles. The complexity of this workflow imposes significant economic burdens, with production costs frequently exceeding $400,000 per dose (Table 1), exclusive of hospitalization and supportive care [37].

In contrast to CAR-T cell therapies, CAR-NK cells and CAR-Ms offer manufacturing paradigms that are inherently more amenable to scalability and standardization, largely due to their suitability for allogeneic, off-the-shelf production. CAR-NK cells can be derived from a variety of sources, including peripheral blood from healthy donors, umbilical cord blood, induced pluripotent stem cells (iPSCs), and established NK cell lines such as NK-92. These sources enable batch manufacturing of large, uniform cell banks, reducing donor-to-donor variability and decoupling product availability from patient fitness or lymphocyte quality. As a result, CAR-NK manufacturing timelines are substantially shorter than those of autologous CAR-T products, often allowing cryopreserved doses to be administered on demand without the need for patient-specific leukapheresis. However, CAR-M production faces its own scalability challenges. The inability of macrophages to expand after differentiation necessitates high-input manufacturing, potentially limiting dose density and increasing per-dose costs.

Overall, the decentralized and manual nature of current manufacturing paradigms limits scalability and reproducibility, posing challenges for widespread clinical deployment. Variability in transduction efficiency, T cell phenotype, and expansion kinetics across patients further complicates standardization and quality control [32]. While allogeneic “off-the-shelf” CAR-T and CAR-NK platforms are under development to circumvent these limitations, they introduce new challenges such as graft-versus-host disease (GVHD), host-versus-graft rejection, and the need for immunoengineering to evade host immune surveillance [38]. These constraints underscore the urgent need for automated, closed-system bioprocessing technologies and universal donor cell platforms to democratize access to CAR-based immunotherapies.

### 2.6. Safety and Toxicity Profile

While CAR-T cell therapy has demonstrated transformative efficacy in hematologic malignancies, its clinical utility is often tempered by the risk of severe and potentially life-threatening toxicities. Foremost among these is cytokine release syndrome (CRS), a systemic inflammatory cascade driven by the rapid activation and proliferation of CAR-T cells upon antigen engagement. This hyper-inflammatory state is characterized by elevated levels of pro-inflammatory cytokines such as IL-6, interferon gamma (IFN-γ), and tumor necrosis factor alpha (TNF-α), and can manifest clinically as fever, hypotension, hypoxia, and multi-organ dysfunction [39]. Management typically involves IL-6 receptor blockade (e.g., tocilizumab) and corticosteroids, though these interventions may attenuate CAR-T cell function and compromise therapeutic outcomes [40]. A second major toxicity is immune effector cell-associated neurotoxicity syndrome (ICANS), which presents with a spectrum of neurologic symptoms including encephalopathy, aphasia, seizures, and, in severe cases, cerebral edema [41,42]. Although the precise mechanisms underlying ICANS remain incompletely understood, current evidence implicates endothelial activation, disruption of the blood–brain barrier (BBB), and neuroinflammation secondary to systemic cytokine diffusion [43]. Additionally, on-target, off-tumor toxicity remains a critical concern, particularly when CARs recognize antigens with low-level expression on healthy tissues, leading to unintended cytotoxicity and collateral tissue damage.

In contrast, CAR-NK cells exhibit a more favorable safety profile, with significantly lower incidences of CRS and ICANS [44]. This is attributed to their innate immune regulation, limited cytokine secretion, and reduced in vivo persistence [45]. However, this attenuated persistence may also contribute to their comparatively diminished anti-tumor efficacy, highlighting the need for strategies that enhance CAR-NK cell expansion and survival without compromising safety. Some studies have investigated co-expansion of CAR-T and CAR-NK cells to mediate this, with some success [46]. These divergent toxicity profiles underscore the importance of precision CAR design and the development of predictive biomarkers to stratify patients by risk and guide clinical management.

In comparison to both CAR-T and CAR-NK cell therapies, CAR-Ms exhibit a distinct and potentially more favorable toxicity profile. To date, clinical and preclinical studies have reported minimal incidence of classical CAR-associated toxicities, including CRS and ICANS, following CAR-M administration [47,48]. This reduced systemic toxicity is largely attributable to the absence of rapid clonal expansion, limited systemic recirculation, and the predominantly localized activity of macrophages within tumor tissues. These observations suggest that CAR-Ms occupy a unique safety niche within the CAR therapeutic landscape and ongoing clinical evaluation will be essential to fully define the therapeutic window of CAR-macrophage-based immunotherapies.

### 2.7. Regulatory and Infrastructure Limitations

The widespread implementation of CAR-immune cell therapies is significantly constrained by regulatory and infrastructural barriers that limit their scalability and equitable global access. The manufacture of these advanced therapies requires Good Manufacturing Practice (GMP)-compliant facilities capable of executing complex workflows, including cell isolation, genetic modification, expansion, and cryopreservation, under rigorous quality control standards. These facilities are capital-intensive and geographically concentrated, resulting in pronounced disparities in access, particularly in low- and middle-income regions where such infrastructure is often lacking or entirely absent [49,50].

The logistical complexity of autologous CAR-T therapy further compounds these challenges. Coordinating leukapheresis, cryogenic transport, and timely reinfusion demands a tightly integrated clinical-manufacturing interface, supported by specialized personnel trained in cell therapy administration and the management of associated toxicities. Moreover, the regulatory landscape for genetically modified cellular products remains fragmented and continuously evolving across jurisdictions. Approval pathways often involve extensive preclinical validation, prolonged review timelines, and stringent post-marketing surveillance, all of which contribute to delays in clinical translation and increased development costs [49,51,52].

Addressing these systemic limitations will require a multipronged approach, including the harmonization of regulatory frameworks, investment in decentralized and automated manufacturing platforms, and global capacity-building initiatives aimed at expanding infrastructure and workforce expertise. For example, the Centre for Regenerative Medicine in Nepal is pursuing the development of cost-effective CAR-T cell therapy by evaluating the feasibility of local production using regionally and internationally sourced reagents [49]. Such efforts are essential to democratize access to CAR-based immunotherapies and ensure their integration into routine clinical practice across diverse healthcare settings.

While CAR-immune cell therapies have revolutionized the treatment of certain hematologic malignancies, their broader application is constrained by multifaceted challenges. These include the immunosuppressive TME, antigen escape, limited persistence, manufacturing hurdles, and safety concerns. Ongoing innovations, such as armored CARs, universal donor platforms, and synthetic biology approaches, aim to overcome these barriers. However, translating these advances into clinically viable, scalable, and globally accessible therapies remains a formidable task.

## 3. The Rise of CAR-Extracellular Vesicles: A Cell-Free Approach

While CAR-based therapies, such as CAR-T and CAR-NK cells, have been successfully used against hematological malignancies, with notable success in relapsed or refractory B cell leukemias and lymphomas, the direct implementation and medical translation have found challenges in cases of poor tumor infiltration, the immunosuppressive TME, inherent antigen heterogeneity, and the risk of severe adverse effects [53,54,55,56,57,58,59,60,61]. Hence, despite the present encouraging results, the challenges aforementioned require alternative approaches that maintain targeting precision while minimizing risks associated with live cell infusion. This has led to the integration of CAR technology with EVs, introducing CAR-EVs, which exhibit superior anti-tumor efficacy compared to their parental live-cell therapies predominantly due to the EVs’ ability to more effectively penetrate solid tumors [62].

EVs have gained attention as next-generation therapeutic delivery vehicles. EV is an umbrella term for lipid bilayer-bound nanoparticles secreted by nearly all cell types, therefore present in nearly all biological fluids, which have been shown to play a critical role in intercellular communication. Exosomes correspond to a subgroup of EVs with highly regulated biogenesis, trafficking, release and uptake processes. Exosomes have an endosomal origin, and their size have been determined to be between 30 and 200 nm. In addition, it has been established that they have biological active content encompassing proteins, lipids, and nucleic acids [63,64] which are transferred to other target cells as part of an exquisite mechanism of the regulation of biological processes [65]. EVs acts as intercellular messengers allowing them to modulate immune responses, mediate tissue repair, and contribute to tumor progression. Increasing interest around EVs is related to their capacity to cross biological barriers such as the BBB, to be inherently biocompatible, and to possess low immunogenicity, making them attractive and ideal candidates for therapeutic engineering applications [66].

### 3.1. Exosome Biogenesis and Cargo-Sorting Mechanisms

**Figure 2:**
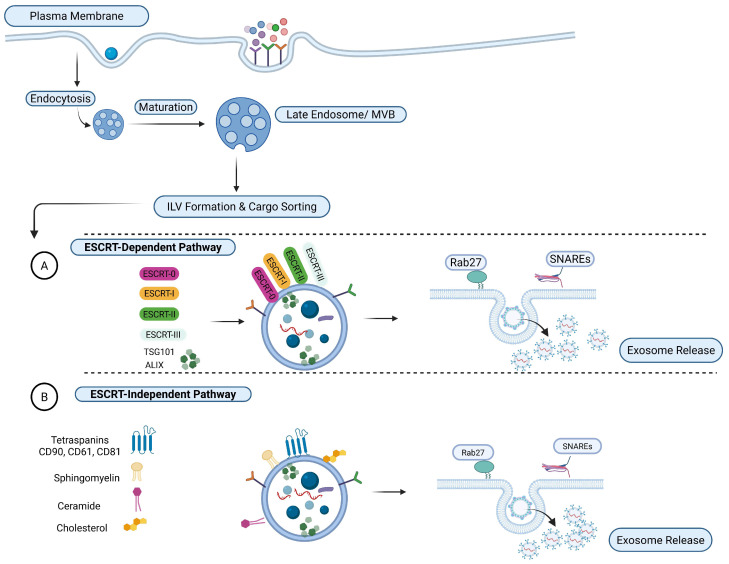
Schematic overview of exosome biogenesis and extracellular vesicle (EV) release. Exosome formation begins with plasma membrane invagination and endocytosis, producing early endosomes that mature into multivesicular bodies (MVBs). Inward budding of the endosomal membrane generates intraluminal vesicles (ILVs) loaded with proteins, lipids, and nucleic acids via both (**A**) ESCRT-dependent (ESCRT complexes, TSG101, ALIX) and (**B**) ESCRT-independent (ceramide, tetraspanins CD9/CD63/CD81) pathways. The ESCRT-0, -I, and -II complexes recognize and sequester ubiquitinated cargo into endosomal membrane domains, while ESCRT-III mediates membrane budding and scission to generate ILVs within the MVB lumen. In parallel, ILV formation can occur independently of ESCRT machinery through lipid- and tetraspanin-driven mechanisms. In this pathway, membrane microdomains enriched in sphingomyelin, ceramide, and cholesterol promote spontaneous inward budding and vesicle formation. The resulting MVBs are either degraded in lysosomes or transported to the plasma membrane, where Rab GTPases and SNAREs mediate the fusion and release of ILVs as exosomes (30–200 nm), which facilitate intercellular communication in physiological and pathological contexts. Created in BioRender. https://BioRender.com/iyn2g1j (accessed on 20 January 2026).

Exosomes are generated through the endosomal trafficking pathway via multivesicular bodies (MVBs), which arise from inward budding of late endosomal membranes to produce intraluminal vesicles (ILVs). During ILV formation, selective membrane-associated and cytosolic cargoes are incorporated through regulated sorting processes. MVBs subsequently either fuse with lysosomes for degradation or with the plasma membrane to release ILVs as exosomes (Figure 2) [67]. The endosomal sorting complex required for transport (ESCRT) machinery plays a central role in ILV formation and exosomal cargo selection. This multi-protein system, comprising ESCRT-0, -I, -II, and -III complexes, coordinates ubiquitinated cargo recognition, membrane deformation, and vesicle scission. ESCRT-0 initiates cargo sequestration, followed by the recruitment of ESCRT-I and -II and the assembly of ESCRT-III, which mediates membrane abscission in a Vps4-dependent manner. The recurrent identification of ESCRT-associated proteins such as TSG101 and Alix in exosomes supports ESCRT-mediated biogenesis, though evidence indicates ESCRT function is not universally required, suggesting cell-type and context-dependent regulation [67,68]. ESCRT-independent mechanisms further contribute to exosome formation through lipid- and tetraspanin-driven membrane organization. Ceramide-enriched micro-domains generated by sphingomyelinase activity promote lateral phase separation and induce negative membrane curvature, facilitating ILV budding while tetraspanins, including CD63, CD81, and CD9, organize micro-domains that selectively recruit protein cargo. Together, these findings support a model in which ESCRT-dependent and -independent pathways coexist to regulate exosome biogenesis and cargo sorting [68,69]. Understanding these biogenesis pathways provides a mechanistic foundation for engineering EVs.

### 3.2. CAR-EVs as a Cell-Free Platform for Targeted Cancer Therapy

In an effort to solve the challenges presented by the cell component of CAR technologies, CAR-EVs represent a hybrid strategy that merges the tumor-specific recognition of CARs with the scalability and safety profile of EVs/exosomes. The strategies developed so far consider the modification of exosomes derived from parent immune cells (e.g., T cells, NK cells, or macrophages) that have been genetically modified to express CAR constructs targeting tumor-associated antigens (TAAs) [70,71,72,73]. As part of the biogenesis process, CAR proteins are incorporated into the EV membrane, enabling antigen-specific binding and in some cases, cytotoxic or immunomodulatory activity [72]. In addition, the modification can occur also at a cargo level by incorporating functional molecules such as granzyme B, perforin, mRNA, or immunoregulatory miRNAs [70,74].

**Figure 3:**
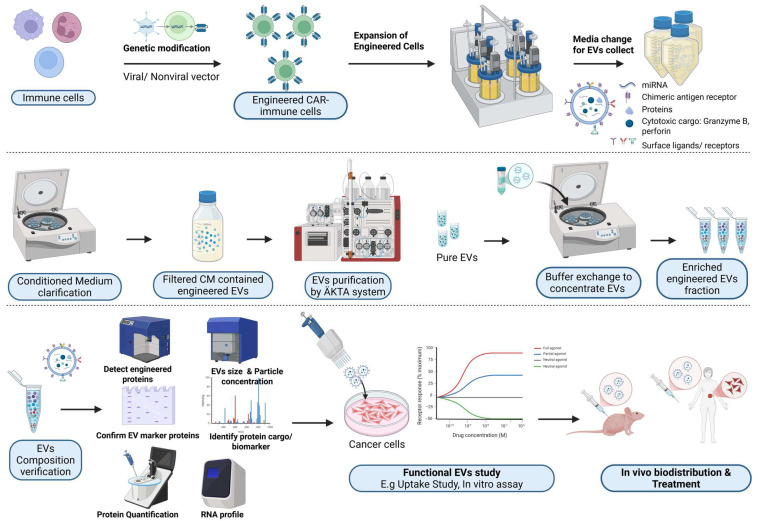
Schematic representation of the workflow for engineering, isolating, and applying immune cell–derived EVs. Immune cells are genetically engineered and expanded in culture to enhance or modify EV production. Conditioned media containing secreted EVs are collected, clarified, and purified using chromatography-based systems (e.g., ÄKTA), followed by buffer exchange to concentrate and obtain highly purified EV fractions. The composition and quality of isolated EVs are validated by flow cytometry (surface marker profiling), nanoparticle tracking analysis/ZetaView (particle size and concentration), Western blotting (protein marker confirmation), and PCR/qPCR (analysis of RNA or DNA cargo). Purified engineered EVs are subsequently assessed in vitro (e.g., uptake and functional assays) and in vivo to evaluate biodistribution, biological activity, and therapeutic potential, supporting their progression toward clinical applications. Created in BioRender. https://BioRender.com/vwahfv2 (accessed on 17 February 2026).

The generation of CAR-EVs typically involves lentiviral or retroviral transduction of donor cells, followed by stimulation to enhance EV secretion (Figure 3). This is then followed by various isolation and purification methods, with the most common approaches being ultracentrifugation, size-exclusion chromatography (SEC), or tangential flow filtration (TFF), which will be further discussed later in the review [75]. Each method carries their own pros and cons, and are highly dependent on the downstream application requirements.

Multiple studies have demonstrated the therapeutic potential of CAR-EVs in preclinical models. For example, engineered CD19-specific CAR-T cells and isolated EVs have been shown to retain tumor-targeting capability and induce apoptosis in CD19+ leukemia cells through granzyme B delivery [70]. Another case demonstrated that EGFR-specific CAR-EVs derived from NK cells suppressed glioblastoma growth and showed efficient biodistribution and tumor homing in murine models [72]. It has also been shown that macrophage-derived CAR-EVs targeting HER2 could stimulate dendritic cell maturation and enhance CD8+ T cell infiltration in HER2+ tumors [76]. These findings underscore the versatility of CAR-EVs not only as direct effectors but also as immunomodulatory agents. Notably, macrophage-derived CAR-EVs (CAR-M-EVs) are under exploration for their ability to activate innate immunity and remodel the TME, a feature less prominent in T or NK cell-derived EVs [71].

## 4. Therapeutic Potential of CAR-EVs

CAR-EVs represent a compelling alternative to traditional CAR-immune cell therapies, offering precise tumor-targeting capabilities while mitigating the safety concerns commonly associated with live cell approaches [77,78]. These vesicles inherit the antigen specificity of their parental CAR-T/NK/M cells, displaying functional CARs on their surface and enabling the recognition of TAAs, including those expressed on solid tumors [70,79]. Their capacity to engage diverse antigens makes them well-suited for addressing the heterogeneity characteristic of many tumor populations. Moreover, due to their nanoscale dimensions, CAR-EVs demonstrate superior penetration into tumor tissues, a notable advantage over bulkier cellular therapies that often face limitations in the infiltration of solid tumors [80,81].

### 4.1. Reduced Toxicity and Increased Safety Profile of CAR-EVs

Currently, the most common toxicities in CAR-T immunotherapy include cytokine release syndrome (CRS) and CAR-T-related encephalopathy syndrome (CRES), with similar toxic effects being observed in other T cell therapies like T cell receptor (TCR) gene therapy and CAR-NK cells [82,83,84]. These adverse events are often linked to the immunosuppressive TME, including inhibitory pathways like PD-1 signaling [85]. CAR-EVs, on the other hand, lack proliferative capacity and do not elicit CRS, offering a safer immunotherapy profile compared to conventional CAR-T therapies [86]. Furthermore, unlike their cellular counterparts, CAR-EVs do not express PD-1, rendering them less susceptible to PD-L1-mediated immunosuppression and preserving their functional integrity within hostile tumor environments [70]. The ability for CAR-EVs to contribute to the anti-tumor immunity extends beyond PD1 and PD-L1 inhibition: they enhance T cell activation, promote cytokine release, and facilitate immune surveillance, potentially establishing long-lasting immunological memory and decreasing the likelihood of tumor recurrence [86,87].

### 4.2. Targeting Specificity of CAR-EVs

The targeting specificity of CAR-EVs is largely inherited from their parent CAR-T cells, which are engineered to express scFvs derived from antibodies. During exosome biogenesis, cellular membrane proteins involved in antigen recognition are often incorporated into the vesicles, allowing CAR-EVs to retain the targeting capabilities of their parental cells. This inheritance of surface molecules has been observed across several cell types; for instance, B cell-derived exosomes have been shown to present peptide-MHC II complexes after internalization and trafficking through MVBs. CAR-EVs have demonstrated tumor-specific targeting and cytotoxic activity in preclinical studies. For example, mesothelin-targeted CAR-T exosomes effectively induced cytotoxic responses in triple-negative breast cancer models by delivering perforin and granzyme B, showing strong anti-tumor efficacy with minimal systemic toxicity [74]. Additionally, exosomes derived from mesenchymal stem cells (MSCs), known for their intrinsic tumor-homing capabilities, further support the feasibility of leveraging natural or engineered targeting traits in EV-based therapies [88,89]. These MSC-derived exosomes mirror their parent cells’ expression of chemokine and growth factor receptors, enabling preferential accumulation at tumor sites.

Of particular interest is their ability to traverse biological barriers such as the BBB, which restricts the entry of ~98% of small-molecule therapeutics and nearly all biologics into the central nervous system (CNS) [90]. Emerging evidence suggests that EVs can effectively bypass both the BBB and the blood–cerebrospinal fluid barrier to facilitate drug delivery to the CNS in various pathological contexts [91,92,93]. This, coupled with their excellent biocompatibility and tissue-specific delivery capabilities, has propelled interest in EVs as therapeutic platforms across a spectrum of diseases, including cancer, neurodegenerative disorders, and inflammatory conditions [94,95].

### 4.3. CAR-EVs as Efficient Therapeutic Delivery Vehicles

In addition to their targeting capabilities, CAR-EVs serve as efficient carriers for therapeutic delivery vehicles. When loaded with chemotherapeutics, CAR-EVs demonstrate enhanced drug accumulation at tumor sites, reduced systemic toxicity, and improved intratumoral penetration, collectively augmenting therapeutic efficacy [96,97,98]. For instance, α-lactalbumin overexpressing EVs engineered to carry Hiltonol, a Toll-like Receptor 3 agonist, and ELANE, a neutrophil elastase, resulted in significant immunogenic cell death and robust CD8+ T cell activation in breast cancer models [99]. Likewise, Shtam et al. demonstrated that electroporation of siRNAs targeting RAD51 and RAD52 in HeLa-derived EVs could induce gene silencing in fibrosarcoma cells, highlighting a novel strategy for disrupting tumor proliferation pathways [100]. Advancement in this field has resulted in several Phase I/II clinical trials currently investigating the potential of EVs as delivery vehicles, highlighting their clinical promise in enhancing therapeutic precision, minimizing systemic toxicity, and enabling targeted treatment strategies [101,102].

Despite the growing evidence supporting CAR-EVs as efficient therapeutic delivery vehicles, precise control over EV cargo composition and selective payload loading remains a major technical and translational challenge. EV biogenesis is a biologically regulated yet inherently heterogeneous process, resulting in vesicle populations that vary considerably in their protein, nucleic acid, and lipid content. Consequently, therapeutic cargo incorporation, whether chemotherapeutic agents, immune modulators, or nucleic acids, often occurs in a semi-stochastic manner, leading to variability in the loading efficiency. Pre-formation loading via photoporation enhanced cargo incorporation into HEK293T-derived EVs (~53% loading for EGFP-targeted nanobodies vs. ~12% for non-targeted dextran), while preserving vesicle size, concentration, and tetraspanin expression [103]. Building on this, Peruzzi et al. (2024) showed that engineering proteins with lipid-raft-associating tags markedly improved selective loading [104]. Proteins were fused to GPI anchors, palmitoylation sites, or transmembrane domains to promote transient association with plasma membrane lipid rafts, resulting in preferential EV incorporation while retaining functional activity. However, overly strong membrane binding reduced delivery efficiency, highlighting the importance of balanced association. Although these strategies were demonstrated with model proteins, they can be directly applied to CAR-EVs. By fusing CAR proteins with lipid-raft-associating motifs, the surface display of functional CARs can be enhanced, selective loading improved, and antigen-targeted activity increased, providing a rational strategy to overcome the inherent cargo-sorting limitations of CAR-EV engineering.

Compared to traditional CAR-immune cell therapies, CAR-EVs offer distinct advantages (Table 2). Their acellular nature eliminates risks of uncontrolled proliferation, CRS, or GVHD [53]. Their small size facilitates deeper penetration into solid tumors and diffusion across dense and intricate extracellular matrices [66,70,71,72]. Additionally, EVs are easier to store, ship, and standardize compared to live cells, making them attractive as “off-the-shelf” products [105,106,107]. Some of these properties lie in their non-replicating ability, their ability to be harvested, purified, and stored as stable biologics and the fact that they can be decoupled from living donor cells once upstream culture is standardized, avoiding the batch-to-batch variation introduced by the use of living cells. From a manufacturing point of view, EVs can be scaled up using bioreactors and cryopreserved without loss of functional activity, simplifying logistics for further clinical translation [75]. In terms of drawbacks, the heterogeneity observed between EV subpopulations needs to be considered for the standardization of a final usable product, as EVs differ in size, cargo composition, and functional activity depending on source cells and culture conditions [105,108]. Specific protocols need to be established for quantifying potency, determining optimal dosages, and developing GMP-compliant manufacturing pipelines, in addition to appropriate quality control processes. Regulatory ambiguity further complicates clinical development: CAR-EVs do not fit neatly into existing categories of biologics or cell therapies, leading to uncertainty about quality control and classification [109,110,111]. Despite these limitations, CAR-EVs have risen as a promising technology for cancer treatment. Moreover, new developments in molecular biology allow engineering of EVs with enhanced tropism, enhanced membrane fusion capability, and cargo-loading precision [112,113,114]. While no CAR-EV therapies have entered clinical trials yet, the expanding body of preclinical data suggests a strong foundation for the first-in-human studies.

## 5. Source and Production of CAR-EVs

**Figure 4:**
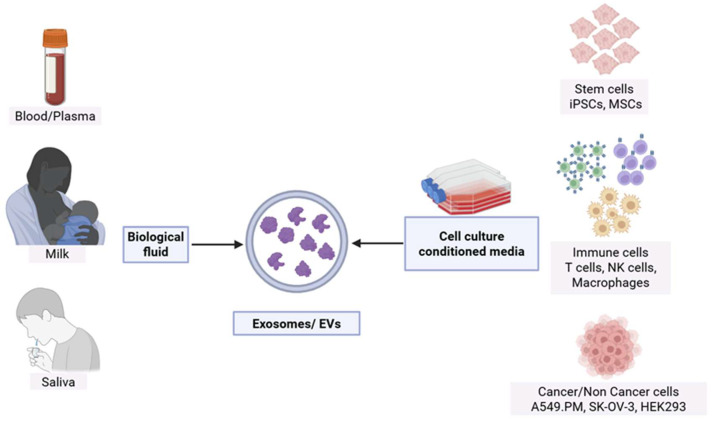
Origins of EVs from both biological fluids and in vitro cell cultures. Biological fluids include blood, urine, saliva, and other bodily fluids, whereas cell culture systems allow EV collection from various cell types, including immune cells, stem cells, and tumor cells. These sources provide diverse EV populations with distinct molecular compositions and functional properties suitable for research and therapeutic applications. Created in BioRender. https://BioRender.com/4cwpfih (accessed on 20 December 2025).

EVs are derived from a wide range of sources, both in vitro and in vivo. In laboratory settings, EVs are commonly harvested from the conditioned media of cultured cells such as immune cells (e.g., T cells, NK cells, macrophages), stem cells, or tumor cells, allowing for controlled production and the possibility of engineering vesicle content or surface markers for therapeutic applications [115]. In comparison, EVs can also be isolated from body fluids, including blood, plasma, urine, cerebrospinal fluid, saliva, and breast milk (Figure 4), where they are naturally released by various cell types throughout the body [115]. These biofluid-derived EVs are highly heterogeneous and reflect the physiological or pathological state of the donor, making them valuable for diagnostic and biomarker discovery [116]. While EVs can be derived from a wide variety of cell types, including mesenchymal stromal cells, tumor cells, and epithelial cells, our discussion is centered on immune cell-derived EVs, specifically those released by CAR-T cells, CAR-NK cells, and macrophages, given their emerging therapeutic and mechanistic relevance.

### 5.1. Engineering Immune Cells for Enhanced Therapeutic Function

The production of CAR-T/NK/macrophage cells using lentiviral vectors begins with the generation of replication-deficient lentiviral particles engineered to carry the CAR transgene and necessary regulatory elements for stable expression. These viral vectors are produced by transfecting packaging cell lines, commonly HEK293T cell, with plasmids encoding the CAR construct along with essential viral structural and packaging proteins. This process results in the assembly and release of lentiviral particles capable of delivering the CAR gene into immune cells, enabling their long-term expression and therapeutic function [117].

The therapeutic potential of CAR-expressing exosomes can be enhanced by optimizing both their release and the expression of CAR molecules on their surface. Strategies such as antigen stimulation using beads coated with recombinant CAR target antigens or co-culturing with cells expressing the relevant antigen have been shown to increase CAR expression on EVs [118]. These enhancements not only improve the targeting ability of CAR-EVs but also amplify their functional potency. Importantly, such optimizations contribute directly to addressing the limitations of conventional CAR-immune cell therapy, particularly in the treatment of solid tumors. Due to their nanoscale size, CAR-EVs can more effectively penetrate dense tumor tissue, a major barrier for full-sized CAR-immune cells. Moreover, their non-proliferative nature mitigates the risk of severe side effects such as cytokine release syndrome and off-target toxicity. CAR-EVs may also exhibit greater resistance to the immunosuppressive TME, further supporting their potential as a safer and more adaptable alternative to traditional CAR-immune cell therapies.

### 5.2. CAR-T Cell-Derived EVs

Patient-derived T cells are isolated from peripheral blood through leukapheresis and subsequently activated in vitro, typically using antibodies against CD3 and CD28, to enhance susceptibility to lentiviral transduction. Once activated, the T cells are exposed to lentiviral vectors, which deliver and integrate the CAR transgene into the host genome, resulting in stable surface expression of the CAR protein. Following transduction, CAR-T cells are expanded ex vivo to achieve therapeutic cell numbers and formulated into a product for infusion. Upon administration, these cells circulate and selectively bind to cancer cells via the scFv domain which mediates antigen specificity by enabling the direct recognition of TAAs, independent of MHC presentation, which then in turn triggers cytotoxic responses and tumor cell elimination [119]. Lentiviral vectors are preferred in CAR-T manufacturing due to their high transduction efficiency in both dividing and non-dividing T cells and their capacity to carry large DNA inserts, allowing the inclusion of complex CAR designs incorporating co-stimulatory and intracellular signaling domains alongside the scFv [119,120].

EVs can be derived from a range of T cell subsets, including cytotoxic T lymphocytes (CTLs), CD4+ T helper (Th) cells, and regulatory T cells (Tregs), each contributing to distinct immunological roles such as immune activation, tumor cell killing, or immunosuppression [73]. In comparison to their parental CAR-T cells, CAR-T cell-derived EVs offer enhanced stability, a defined lifespan, and an inability to proliferate. These properties make CAR-T EVs a potentially safer immunotherapeutic alternative, with a lower risk of off-target toxicity and immune-related adverse effects [118].

EVs derived from T-lymphocytes express TCR and proapoptotic molecules, endowing them with both antigen specificity and cytotoxic functionality. This indicates that EVs derived from CAR-T cells may serve as effective vehicles for delivering proapoptotic signals directly to cancer cells, offering a novel cell-free immunotherapeutic approach. The release of T cell-derived EVs is closely associated with the formation of an immune synapse (IS). This process is initiated when TCRs engage with antigens or MHC molecules on the surface of antigen-presenting cells. In response, CD4+ helper T cells become activated and secrete cytokines, while CD8+ CTLs are primed to kill target cells. During IS formation, secretory granules and MVBs polarize toward the synapse, facilitating the targeted release of EVs, including exosomes [73,118]. CTL-derived exosomes are particularly enriched with cytotoxic proteins such as perforin and granzymes, along with surface molecules including TCR and CD8, enabling them to mediate antigen-specific killing with minimal off-target effects. These exosomes also carry proapoptotic ligands like Fas ligand (FasL) and Apo2 ligand (Apo2L), which can induce apoptosis in both target and effector T cells, contributing to immune regulation through activation-induced cell death (AICD) [73,118].

### 5.3. CAR-NK Cell-Derived EVs

NK cells, specialized killer cells, are types of innate immune cells with the ability to lyse tumor cells without MHC restriction. The human body contains approximately 20 to 50 billion NK cells in total. In the blood, NK cells make up 5–15% of lymphocytes, with about 0.5 to 3 billion circulating at any time. The rest are distributed across tissues like the liver, lungs, spleen, bone marrow, and lymph nodes, where they play key roles in immune surveillance [121]. While cord blood-derived CAR-NK cells have shown promise, sourcing NK cells from peripheral blood poses practical challenges due to difficulties in isolation, purification, expansion, and genetic modification [122]. To overcome these limitations, immortalized NK cell lines offer a viable and scalable alternative. Among these, the NK-92 cell line, developed from a lymphoma patient and available from the American Type Culture Collection (ATCC, USA), has emerged as a widely used model. NK-92 cells can proliferate indefinitely, provide a homogeneous and easily expandable population, and are amenable to genetic engineering. Notably, CAR-engineered NK-92 cells have demonstrated robust anti-tumor activity, making them a practical and potent platform for both cell-based and EV-based immunotherapies [123]. Studies on CD19+ lymphoid tumors have shown that CAR-NK cells derived from cord blood can provide strong anti-tumor responses [124,125]. These engineered cells, often supplemented with IL-15 to improve their persistence and expansion, have demonstrated the capacity to induce rapid and complete remissions in patients, with minimal adverse effects [125].

The generation of genetically modified CAR-NK cells relies on two major strategies: viral transduction and non-viral transfection. Viral vectors, particularly retroviruses and lentiviruses, have been widely used due to their ability to stably integrate transgenes into the NK cell genome [126]. While retroviruses offer high integration efficiency, they require actively dividing cells and pose risks such as insertional mutagenesis [127]. On the other hand, lentiviruses, which can accommodate larger transgenes (up to 10 kb) and exhibit lower insertional risk are preferred. However, they are limited by their transduction efficiency (often below 10%) partly due to limited expression of the low-density lipoprotein receptor (LDLR) for the commonly used vesicular stomatitis virus (VSV) encoding glycoprotein (VSV-G) envelope [128,129]. Modified pseudo-types and pharmacological interventions, such as statins and geranylgeranyl-pyrophosphate (GGPP), have also been explored to enhance viral entry and maintain NK cell cytotoxicity [130].

Non-viral approaches, including mRNA electroporation and lipofection, are gaining traction due to their transient expression profiles, lower cytotoxicity, and compatibility with GMP manufacturing, with mRNA electroporation achieving up to 95% transfection efficiency in activated NK cells [131]. However, the transient nature of CAR expression with these approaches limits their therapeutic duration, often requiring repeated administrations. To address this, transposon-based systems have emerged as non-viral alternatives capable of stable gene integration. These systems utilize a transposase enzyme to insert gene-bearing transposons, such as CAR constructs, directly into the genome, enabling long-term expression [132]. Despite their promise, the application of transposon technologies in primary NK cells remains technically demanding. Recently, CRISPR/Cas9 gene editing has emerged as a powerful tool that enables precise CAR integration at defined genomic loc. This approach also allows for functional enhancements, such as the deletion of inhibitory receptors (e.g., NKG2A) or the incorporation of safety switches [133]. However, current knock-in efficiencies in primary NK cells remain relatively low (3–16%), underscoring the need for continued optimization [134]. Each of these strategies presents unique advantages and challenges in terms of stability, efficiency, safety, and scalability, and ongoing research aims to refine these technologies to support robust, clinically viable CAR-NK cell manufacturing [135]. There is growing scientific interest in CAR-NK cell-derived EVs over those derived from CAR-T cells, largely due to their advantages in safety, scalability, and therapeutic potential. A primary concern with CAR-T cell therapies, and by extension, their EVs, is the risk of severe adverse effects, including CRS and neurotoxicity [136]. In contrast, CAR-NK cells and their EVs present a significantly lower risk of such toxicities [137]. Additionally, CAR-NK EVs often retain the innate tumor-killing abilities of NK cells and are less affected by the immunosuppressive TME, enhancing their potential effectiveness against solid tumors. These EVs can also deliver cytotoxic proteins like perforin and granzymes, as well as regulatory microRNAs, directly to tumor cells [138]. Finally, NK-derived EVs are generally more stable, easier to handle, and less prone to issues like cellular exhaustion, making them attractive for clinical application [139]. Taken together, these factors make CAR-NK EVs a promising and potentially safer alternative to CAR-T EVs in the development of next-generation cell-free cancer immunotherapies [140]. Given the unique benefits of CAR-NK-derived EVs, it is plausible to expect that CAR-NK-EVs might also deliver powerful anti-tumor effects while maintaining a favorable safety profile. Further studies are needed to explore whether CAR-NK-EVs, particularly those incorporating strategies like IL-15 for enhanced persistence, could match or even exceed the clinical outcomes seen with the parent CAR-NK cells.

### 5.4. CAR-Macrophage-Derived EVs

Macrophages, a subset of leukocytes derived from circulating monocytes, are essential components of the innate immune system, maintaining tissue homeostasis, regulating inflammatory responses, and defending the host against a variety of pathogens and diseases [141]. Beyond their established roles in immune surveillance, macrophages exhibit remarkable functional plasticity, enabling them to adopt distinct phenotypes in response to environmental cues [142]. Within TME, this adaptability results in a dual, context-dependent role: macrophages can either inhibit or promote tumor progression depending on their polarization state, rendering them a double-edged sword in cancer biology [143].

The classical M1 phenotype, induced by IFN-γ or lipopolysaccharide (LPS), is pro-inflammatory and exerts potent anti-tumor activity through the secretion of cytokines such as TNF-α and IL-12, as well as reactive oxygen and nitrogen species. In contrast, the alternatively activated M2 phenotype, stimulated by IL-4, IL-10, or TGF-β, is anti-inflammatory and supports tissue repair, angiogenesis, and immune suppression [143]. Within the TME, macrophages are exposed to tumor-derived signals that predominantly drive them toward an M2-like state, forming TAMs. These TAMs promote tumor growth, metastasis, and immune evasion by secreting pro-tumor cytokines, matrix-remodeling enzymes, and angiogenic factors [143]. Importantly, TAMs retain a degree of plasticity and can, under specific stimuli, transition toward an M1-like phenotype, thereby exerting anti-tumor effects [144].

This functional heterogeneity is reflected in the EVs released by macrophages, which carry proteins, lipids, and RNAs characteristic of the parent cell’s polarization. These EVs influence recipient cells within the TME, transmitting either anti-tumor or pro-tumor signals. M1-derived EVs (M1-EVs) typically display anti-tumor activity, being enriched with pro-inflammatory cytokines, microRNAs such as miR-155 and miR-125b, and other bioactive molecules that can induce apoptosis in cancer cells, inhibit proliferation, and stimulate anti-tumor immune responses. Notably, M1-EVs can also reprogram TAMs toward an M1-like phenotype, amplifying cytotoxic and immune-stimulatory effects within the TME [145]. Conversely, M2-derived EVs (M2-EVs) generally promote tumor progression. They contain immunosuppressive cytokines, pro-angiogenic factors, and microRNAs such as miR-21 and miR-29a, which enhance tumor cell proliferation, invasion, metastasis, and immune evasion. M2-EVs can polarize other macrophages toward an M2 phenotype, reinforcing a pro-tumor TME [145].

Given the pivotal role of macrophage plasticity and EV-mediated communication in tumor progression, recent advances in nanotechnology have sought to exploit these mechanisms for therapeutic benefit. For example, Sono@NAT10 nanorobots have been developed to target NAT10 condensates in macrophages, promoting the M2-to-M1 transition, enhancing anti-tumor immunity and suppressing tumor growth [146]. In vivo studies demonstrated that Sono@NAT10 improves the efficacy of immunotherapy, representing a novel strategy for the precise manipulation of macrophage function within the TME. Despite these promising results, translation to human therapy faces challenges: mouse models may not fully recapitulate human colorectal cancer, and further studies are needed to evaluate the safety, efficacy, and precision of the ultrasound-triggered release system, as well as its adaptability across different tumor types.

Building on the concept of macrophage-targeted nanotherapy, other strategies have similarly leveraged engineered macrophage-derived vesicles to enhance anti-tumor immunity. Engineered EVs offer a cell-free therapeutic platform, capable of delivering anti-tumor cargo or reprogramming TAMs while circumventing limitations associated with live-cell therapies, such as safety concerns, tissue penetration, and manufacturing complexity [145,146]. A striking example of this approach is provided by Yuchen et al., who developed a method for in situ generation of CAR-Ms using inhaled, engineered small EVs (sEVs). These CARmRNA@aCD206 sEVs were produced by transducing 293T cells with a lentiviral construct and employing a modified EXOtic device to encapsulate mesothelin (MSLN)-specific CAR mRNA. The vesicles were further engineered to express CD63-L7Ae and display anti-CD206 scFv, allowing targeted delivery to CD206+ macrophages. Inhalation facilitated preferential accumulation of sEVs in lung tissue, where they delivered CAR mRNA to macrophages, generating functional CAR-Ms in situ. These CAR-Ms exhibited enhanced phagocytosis, immune activation, and TME reprogramming, leading to significant tumor inhibition, prolonged survival, and durable immune memory, even in heterogeneous tumors lacking the original CAR antigen. This approach exemplifies a safe and effective platform for macrophage-based immunotherapy, overcoming the limitations of conventional ex vivo CAR-M generation [147]. Collectively, these studies illustrate a growing trend in nanotechnology-driven macrophage reprogramming, where both targeted nanorobots and engineered EVs are used to modulate TAMs and enhance anti-tumor immunity. Such strategies highlight the therapeutic potential of exploiting macrophage plasticity within the TME, offering new avenues for cancer immunotherapy while minimizing the challenges associated with traditional cell-based therapies.

While macrophages highlight the role of immune cells in shaping the TME through EVs, other immune cell types, including dendritic cells, T cells, B cells, and NK cells, also secrete EVs with therapeutic potential. These EVs carry molecular and functional signatures of their parent cells, making them versatile platforms for engineering and targeted therapy. Advances in immune cell engineering, culture expansion, and EV purification now enable the production of tailored EVs suitable for preclinical testing and eventual clinical translation, which will be further discussed in upcoming sections.

## 6. CAR-EVs in Hematological Malignancies (Preclinical and Clinical Studies)

With the first breakthrough of a successful CAR treatment for patients with B cell lymphoma in 2010, hematological malignancy has been one of the major focuses in the development of CAR-cell therapy [148]. Since then, CAR-cell therapies have been developed to target various hematological malignancies including non-Hodgkin’s lymphoma, B cell acute lymphoblastic leukemia, and multiple myeloma and to date [149], with FDA having approved six CAR-T cell products for hematological malignancy indication (Table 1). Primarily, these therapies have been designed to target antigen such as CD19, which has been noted as one of the most common target antigens in hematological malignancies. Despite its rising popularity, the use of CAR-cell therapy has been restricted by several factors, such as the risk of CAR-T cell exhaustion and CRS which may result in complications post-treatment [150]. As such, the focus of the CAR-related therapy development has shifted towards a cell-free approach, with EVs being an attractive candidate for cancer therapy.

As previously discussed, EVs offer an advantage over cell therapy as they are immune to suppression by the PD1 pathway [70,86]. This advantage has been observed in preclinical studies where the expansion and the anti-tumor effects of CAR-T cell were enhanced following pre-treatment with CD19-expressing EVs, suggesting that CAR-derived EVs can promote cytotoxicity and anti-tumor effects of the treatment [151]. Another piece of evidence of anti-tumor properties of CAR-EVs in hematological malignancies was observed in a study by Haque and Vaiselbuh in 2010, where the pair transduced HEK293T cells with plasmid construct expressing CD19-CAR and isolated the EVs (denoted Exo-CD19 CAR) from cell conditioned media to treat CD19+ leukemia B cells in culture [152]. Assessment of cell death showcased not only the efficacy, but also the specificity of CAR-EVs as it was shown that only the viability of leukemia B cells that expressed CD19 were decreased following co-culture with Exo-CD19 CAR.

Most recently, Lanuti and colleagues demonstrated that CAR-EVs help predict the persistence of CAR-T cells in the circulatory system 2 years following administration. In the 2-year cohort study involving 22 patients receiving CD19-targeting CAR cell therapy, it was observed that circulatory CD19+ CAR-T cell-derived EVs (CD19.CAR + EVs) was noticeably decreased in recipients who did not respond to treatment, making EVs a powerful indicator of the efficacy of CAR therapy. Furthermore, using functional assays the team demonstrated a dose-dependent cytolytic activity of the CD19.CAR + EVs in two different cell lines of hematological malignancy, highlighting the therapeutic potential of CAR EVs [139].

In a clinical setting, there has only been a single clinical trial (NCT03608631; often referred to as the *iExosomes* trial) exploring the use of EVs in metastatic pancreatic cancer patients. In >90% of pancreatic ductal adenocarcinoma (PDAC) cases, the Kirsten rat sarcoma viral oncogene homolog (KRAS) gene is mutated, with the G12D mutation being one of the most common oncogenic variants [153]. In this most novel study involving the use of MSC-derived exosomes, patients received intravenous (IV) infusion of exosomes loaded with a small interfering RNA (siRNA) against KRAS G12D. Initial findings from the trial indicate that this therapeutic approach demonstrates signs of target engagement and immune modulation while appearing to be safe; however, longer-term studies with larger cohorts are required to draw more conclusive statements regarding its efficacy and clinical benefit.

While limited trials have directly investigated the anti-tumor effects of EVs, CAR-EVs have been identified as an indicator of systemic toxicity. A previous finding suggests that assessing levels of CAR-released EVs in plasma can help predict the risk of ICANS incident in B cell lymphoma patients who received anti-CD19 CAR-T therapy, as it was observed that increased levels of circulating CAR-EVs correlated with increased risk of ICANS [154]. However, there is still very limited number of in vitro and in vivo studies which explore the roles of CAR-EVs in hematological malignancies, making it difficult to draw conclusive statements. Nevertheless, the current available studies were able to show snippets of CAR-EVs potential as a treatment option for hematological malignancy as well as highlighting its advantages over conventional CAR-immune cell therapy.

## 7. CAR-EVs in Solid Tumors (Preclinical and Clinical Studies)

The use of CAR-EVs in the treatment of solid tumors is an emerging field, presenting possibilities of accessing parts of the tumor where other treatments are likely to struggle. EVs are biologically derived, advantageously carrying surface markers akin to cells hence minimizing immunogenicity of these particles. In addition, unlike CAR-T/NK cells, EVs are significantly smaller in size, enabling infiltration into the tumor core where access often presents a challenge. Unlike small molecules, EVs can be engineered with exterior domains comprising targeting molecules with an affinity to surfaces of tumor cells, encouraging specific uptake into such cells.

### 7.1. Breast Cancer

Of note, research around the use of CAR-EVs in breast cancer is on the rise. CAR-EVs derived from EGFR and HER2 targeting CAR-T cells were shown to specifically target EGFR and HER2 overexpressing cells respectively, decreasing tumor proliferation and growth in vitro and in vivo [70]. A groundbreaking study introduced the biomimetic Exo_CAR/T7_@Micelle nanoplatform, a cutting-edge drug delivery system designed to target breast cancer brain metastases. This innovative approach successfully transported stealth cargo, releasing RSL (a ferroptosis inducer) directly into metastatic cells, triggering potent anti-tumor activity [155]. In another study, Yang and colleagues focused on targeting mesothelin in triple-negative breast cancer, resulting in a decrease in tumor growth attributed by the presence of cytolytic cargo such as granzyme B and perforin [74]. Furthermore, in a Phase I trial (NCT03545815), the use of mesothelin-targeting CAR-T cells engineered using CRISPR-Cas9 to downregulate PD-1 and TCR expression, demonstrated the persistence of these cells, enhancing treatment potency [156]. Whilst there are no clinical trials specifically relating to the use of CAR-EVs in breast cancer, their therapeutic potential presents an attractive avenue for future clinical breakthroughs.

### 7.2. Ovarian Cancer

In ovarian cancer, the use of CAR-EVs remains to be explored. Research involving the use of CAR cell therapy in ovarian cancer is on the rise, with studies investigating their possibility for targeting overexpressed biomarkers unique to signatures of ovarian cancer disease progression. CAR-T cells designed to target TAG-72 and CD47 successfully eliminated recipient ovarian cancer cells, while minimizing non-specific tissue damage due to the ubiquitous expression of CD47 in various normal cell types [157]. Recently, a Phase I clinical trial (NCT05316129) utilizing follicle-stimulating hormone receptor (FSHR) targeting autologous CAR-T cells has been initiated for patients with recurrent platinum resistant or refractory ovarian cancer (OVCA). Recent advancements in EV research have demonstrated that precision targeting of ovarian cancer cells can be significantly improved through strategic engineering of EVs designed to selectively bind to key molecular markers, including EphB4 and ephrinB2 [158,159]. These findings emphasize the potential of EV-based therapeutic approaches in enhancing targeted cancer treatment and improving clinical outcomes.

### 7.3. Lung Cancer

Currently, the use of CAR-EV as therapy in lung cancer also remains at a preclinical research phase. The use of CAR-T EVs as carriers for paclitaxel (PTX@CAR-Exos), a commonly used chemotherapeutic agent, demonstrated promising results when administered as an inhalable form in vivo [97]. In terms of clinical trials, CAR-T/NK cells are a major focus, offering various approaches to targeting specific lung cancer subtypes. Promising targets include MUC1 and PD-1 in non-small cell lung cancer (NCT03525782), DLL3 in small cell lung cancer (NCT05507593 and NCT06348797) and carcinoembryonic antigen (CEA) in CEA-positive advanced lung cancer (NCT06768151). The use of dual-targeting CAR, EGFR/B7H3 CAR-T in lung cancer (NCT05341492) is in progress, with some of these targets common in other cancers (e.g., breast cancer, glioblastoma), offering opportunities for interchangeable use across multiple indications.

### 7.4. Glioblastoma

Glioblastoma (GBM) is one of the most aggressive and treatment-resistant cancers. Traditional therapies, including surgery, radiotherapy, and chemotherapy, offer limited efficacy primarily due to the tumor’s ability to infiltrate the surrounding brain tissue, its heterogeneous nature, and the BBB that limits drug delivery. As a result, there is an urgent need for new therapeutic strategies to treat GBM more effectively. While CAR-T/NK cells present a promising option, challenges such as limited accessibility to tumors located in the brain prevent it from being able to treat solid tumors like GBM. Hence, the use of EVs derived from CAR-T and CAR-NK cells have emerged as an alternative potential.

Preclinical studies have demonstrated that CAR-T exosomes retain the tumor-targeting capabilities of their parent cells, while showing reduced immunogenicity and toxicity. For instance, exosomes from CAR-T cells targeting EGFRvIII have shown cytotoxicity against GBM cells by delivering cytolytic proteins such as perforin and granzyme B directly to the TME. EVs can bypass the immunosuppressive barriers and potentially cross the BBB due to their small size, which makes them a great potential candidate for GBM therapy [160]. Similarly, exosomes from CAR-NK cells have been found to induce apoptosis in glioblastoma models and reduce tumor growth in vitro and in vivo [161].

While no clinical trials are investigating the applications of CAR-EVs in glioblastoma to date, there is an ongoing Phase I clinical trial (NCT03383978) evaluating HER2-targeting CAR-NK cells in recurrent GBM. With potential positive findings from the clinical trial and encouraging results for CAR-derived EVs in the treatment of glioblastoma in preclinical settings, future extensions could explore EVs as a less toxic delivery modality in a clinical setting.

### 7.5. Pancreatic Cancer

Pancreatic ductal adenocarcinoma (PDAC) remains one of the most lethal malignancies, largely due to its aggressive nature, late diagnosis, and resistance to conventional therapies. Preclinical studies have demonstrated the efficacy of CAR-EVs in reducing tumor size in an aggressive orthotopic murine PDAC model [162]. Although no clinical studies have yet investigated the application of CAR-T/NK cell-derived EVs in pancreatic cancer, there is growing evidence supporting the efficacy of CAR-T and CAR-NK cell therapies in this context. For instance, preclinical models using mesothelin-targeted CAR-T EVs demonstrated efficient and selective killing of mesothelin-positive pancreatic cancer cells both in vitro and in murine xenograft models [163]. Additionally, in a clinical trial (NCT03941457) evaluating the Robo1-specific CAR-NK, the team demonstrated that a pancreatic lesion and liver metastasis in a patient were controlled within 5 months of administering the treatment [164]. While somewhat positive results were observed, the authors did acknowledge that the efficacy of NK cell therapy on solid tumors was limited due to the immunosuppressive TME and the inability of NK cells to enter the tumor tissue given its size. Given that EVs possess enhanced tissue penetration and a reduced risk of eliciting adverse immune responses compared to cellular therapies, they represent a promising alternative approach in the setting on pancreatic cancer. Future studies should consider evaluating the therapeutic potential of CAR-T and CAR-NK EVs in pancreatic cancer.

Thus far, we have explored the use of EVs derived from CAR-NK and CAR-T cells in treating lung, breast, ovarian, pancreatic, and glioblastoma cancers. While preclinical and clinical studies investigating applications of CAR-engineered immune cell-derived EVs in other solid tumors are limited, several studies have shown potential of CAR-engineered immune cells in several other cancers including colorectal, prostate, gastric, head and neck squamous cell carcinoma, and hepatocellular carcinoma [165,166,167,168,169]. Considering the results from these findings and the advantages of EV over cell therapy covered earlier in the review, future extensions of these cell-based studies could explore EVs as a less toxic and more efficacious therapeutic option.

## 8. Large-Scale Isolation and Purification of CAR-EVs

EVs, particularly CAR-EVs, are emerging as potent therapeutic agents for targeted drug delivery and immune modulation [62]. However, moving CAR-EVs from research into clinical use depends not only on their potential benefits, but also on the ability to isolate them at a scale suitable for clinical or in vivo therapeutic use. Therapeutic doses of EVs are often considerably large (commonly between 10^10^ and 10^14^ particles per dose) due to challenges with systemic distribution and the quantities required for efficacy [170,171]. This is in part due to in vivo pharmacokinetics of EVs being poorly characterized, representing a critical knowledge gap for clinical translation. Quantitative studies have repeatedly shown that circulatory half-life is generally very short, with systemic administration in rodents showing an initial rapid distribution phase (T_1_/_2_α) of the order of ≈1.5–20 min and a slower elimination phase (T_1_/_2_β) of ≈35–185 min, depending on labeling method, source, and detection sensitivity [172]. Several groups have revealed that unmodified EVs are typically detectable for less than 30 min in circulation in small-animal models before becoming sequestered in the liver and spleen, with very limited active delivery to distal tissues [173,174]. In contrast, studies in non-human primates suggest that EV persistence in plasma may be longer (e.g., ~36–42 min in early decay phase, with some signal detectable up to 24 h depending on dose and model), highlighting potential interspecies variation in clearance rates [175]. These rapid systemic clearances explain why only a small fraction of an intravenous dose ultimately reaches tumors or other target sites. In addition to circulation time, stability and potency of EV-associated cargos are major determinants of functional delivery. In light of these pharmacokinetic constraints, the field has increasingly prioritized strategies to enhance EV production yield, aiming to mitigate the challenges posed by short half-life and limited in vivo stability.

These demands place substantial pressure on downstream workflow to deliver yield, purity, and biological activity of CAR-EVs at industrial scales. Importantly, EV isolation methods differ substantially in recovery yield, co-isolated impurities, and preservation of functional surface proteins, and these differences can influence downstream functional conclusions; however, this section focuses specifically on isolation platforms with demonstrated scalability and relevance to clinical translation. An ideal isolation strategy must address several interdependent constraints: recovery yield, purity, batch-to-batch reproducibility, scalability to multi-liter or bioreactor production volumes, buffer compatibility, and preservation of vesicle integrity [176]. Furthermore, the method must integrate seamlessly into a broader manufacturing pipeline that includes upstream bioreactor production and downstream formulation, storage, and release testing [177]. Finally, the method intended for clinical or commercial use must be suitable for current GMP requirements or readily adaptable to future GMP integration. Among the various platforms under consideration, TFF and chromatographic techniques have emerged as the most practical and scalable solutions for large-scale EV production [176].

### 8.1. Tangential Flow Filtration (TFF)

TFF is a membrane-based technique that separates particles by size as the fluid flows tangentially across the filter surface [178]. In contrast to dead-end filtration, TFF reduces fouling and enables continuous, large-volume processing [179]. It is widely used upstream of chromatography for bulk concentration, buffer exchange, and diafiltration of EV harvests [176,180,181,182,183]. Despite its advantages, TFF presents certain operational considerations. Despite being less prone to fouling, pore clogging can still occur, especially with complex media or high particle loads [179]. More critically, TFF lacks molecular specificity and may co-isolate protein aggregates, lipoproteins, or other macromolecular contaminants [182]. Additionally, improper control of transmembrane pressure or flow rate can cause shear-induced damage to vesicles, which is especially problematic for CAR-EVs due to their functional surface protein display [75]. To overcome this limitation, chromatography is commonly employed downstream to refine the EV population and improve product quality.

### 8.2. Chromatographic Purification

Chromatography has become an established method for EV isolation, widely adopted across research and industrial workflows. Its proven specificity, scalability, and adaptability make it a central platform currently employed in large-scale purification processes for EV manufacturing [183] and a promising tool for CAR-EVs production. Techniques such as SEC, affinity chromatography, ion exchange chromatography (IEX), and multimodal chromatography (MMC) have been adapted and shown varying degrees of success in scaling up EV isolation [184,185,186]. A comparison of these techniques is summarized in Table 3. These methods are often implemented using automated platforms, such as Cytiva’s ÄKTA fast protein liquid chromatography (FPLC) system, to enable scalable, reproducible, and GMP-compatible processing.

The selection of a chromatographic strategy is typically guided by its balance of selectivity, throughput, cost, and compatibility with upstream TFF-based enrichment. While each chromatographic method offers unique benefits, successful implementation requires attention to certain downstream processing considerations. For example, many chromatographic techniques, such as SEC and IEX, elute EVs over relatively large volumes, resulting in product dilution. This necessitates an additional volume reduction step, especially for applications requiring high-dose or concentrated formulations [187,188]. Additionally, elution buffers optimized for chromatographic resolution may not be physiologically compatible, requiring downstream buffer exchange via diafiltration or TFF prior to final formulation. These process interdependencies highlight the importance of integrated workflow design, where chromatography conditions are harmonized with both upstream production and downstream clinical requirements.

**Table 3:** Summary of chromatographic modalities used in large-scale EV purification.

Method	Principle	Advantages	Limitations	Industry Use Examples
Size-Exclusion Chromatography	Size-based separation via porous matrix	Gentle on EVs; preserves structure; scalable with FPLC; GMP-compatible	Broad elution profiles cause dilution; limited column capacity lowers throughput at industrial scale	Cytiva (HiScale), Marlborough, MA, USA
Affinity Chromatography	Ligand-specific binding to surface markers	High specificity; enables selective enrichment; retains biological function	Expensive ligands; limited resin reusability; lower flow rates; challenging scale-up	FUJIFILM Wako (MassivEV™, Tim4 functionalized columns), Osaka, Japan [189]
Ion Exchange Chromatography	Charge-based interaction with resins	Cost-effective; scalable; good impurity removal; high binding capacity GMP-compliant workflows	Requires precise control of pH and ionic strength; risk of EV loss if conditions not optimized	Codiak BioSciences, Cambridge, MA, USA [190]
Multimodal Chromatography	Mixed-mode, notably size-exclusion, ionic, and hydrophobic interactions	Handles complex harvests; robust impurity clearance; good for serum-rich media	Requires empirical method development; complex elution conditions; higher resin cost	RoosterBio, Frederick, MD, USA in collaboration with Cytiva, Marlborough, MA, USA (Capto™ MMC, Capto™ Core 700)

### 8.3. Integration and Operational Trade-Offs

In current EV manufacturing pipelines, chromatography is commonly paired with TFF in a sequential, integrated workflow. TFF is typically used as the initial step for bulk concentration and diafiltration of bioreactor harvests, which enables chromatography to operate more efficiently on concentrated, lower-impurity inputs. This configuration, TFF followed by SEC, IEX, or affinity capture, has been widely adapted for both preclinical and GMP-aligned EV production. Academic studies have adopted this sequential approach to ensure yield and reproducibility for in vivo studies [178,180,191]. Likewise, commercial manufacturers, such as Esco Aster and Creative Biolabs use TFF to pre-process bioreactor harvests prior to chromatography purification. For example, Cytiva has proposed integrated workflows using TFF and SEC for EV production from cell culture supernatants of HEK293 cells while RoosterBio in collaboration with Cytiva is known to use TFF to reduce bioreactor harvest volumes before applying either SEC or MMC as polishing steps. FUJIFILM Wako’s MassivEV™ platform represents an advanced application of this model, using TFF for volume reduction followed by Tim4-affinity chromatography for high-specificity EV capture.

From an operational perspective, this integration also balances time and cost. TFF systems are well-suited for processing large volumes of harvest material and are often more cost-effective than chromatography, with membrane costs significantly lower than chromatography resins. Chromatography, while essential for clinical-grade purity, poses throughput limitations due to slower flow rates and higher per-use costs. Resins used in affinity and MMC modalities are expensive and susceptible to fouling or ligand degradation. Moreover, while TFF scale-up is relatively linear via membrane surface area expansion, chromatography scale-up is more complex, requiring parallel columns or specialized hardware such as high-flow FPLC systems. These operational trade-offs have led to a widely adopted configuration in large-scale workflows, where TFF serves as the primary isolation step to concentrate and clarify crude harvests, followed by chromatography as a purification step to achieve clinical-grade quality.

The integration of TFF and chromatography thus represents the most practical and widely adopted model for large-scale CAR-EV purification. It satisfies critical criteria, including yield, purity, structural integrity, and GMP compatibility while allowing for flexible adaptation to different production scales and regulatory contexts.

## 9. Challenges and Future Directions

While CAR-EVs hold immense promise, challenges remain. Ensuring their scalability, safety, and long-term efficacy demands rigorous research. Additionally, understanding their interactions with the immune system, tumor heterogeneity, and personalized approaches will shape their clinical translation. Thus far, in this review, we have explored the multifaceted world of CAR-EVs, dissecting their therapeutic potential, immunogenicity, and role in reshaping cancer treatment. However, realizing this promise requires addressing a range of scientific, manufacturing, and regulatory hurdles.

### 9.1. Manufacturing and Scalability

One of the foremost challenges is the scalable and reproducible manufacturing of CAR-EVs under GMP conditions, and this remains a key bottleneck in bringing CAR-EVs to the clinic. Unlike traditional biologics, CAR-EVs are complex, nanoscale vesicles derived from engineered donor cells, and their production involves multiple tightly controlled steps, from parental cell line engineering and expansion to vesicle isolation, purification, and formulation. The heterogeneity of EVs, variability in cargo loading, and sensitivity to processing conditions make it difficult to achieve batch-to-batch consistency at the clinical scale. Furthermore, conventional isolation methods such as ultracentrifugation or density gradient separation are labor-intensive and poorly scalable, while newer techniques like TFF or SEC are promising but require further standardization and validation for clinical-grade applications. Additionally, ensuring compliance with GMP requires robust in-process controls, validated cleanroom procedures, and well-defined release criteria, all of which are still evolving for EV-based therapeutics. The development of closed, automated manufacturing platforms and standardized EV production protocols will be crucial to enabling broader clinical translation and regulatory approval.

### 9.2. Characterization and Quality Control

The comprehensive characterization of CAR-EVs remains a critical unmet need. Current technologies often fall short in accurately defining vesicle size distribution, surface markers, cargo composition, and potency. Regulatory agencies such as the FDA and EMA have yet to establish formalized quality control frameworks specific to EV-based therapeutics. In the absence of standardized reference materials or potency assays, ensuring product consistency and predictability across batches is challenging.

### 9.3. Immunogenicity and Safety

Though CAR-EVs are considered less immunogenic than whole-cell therapies, their long-term safety profiles remain to be fully interpreted. Risks of off-target effects, unintended biodistribution, or the transfer of oncogenic or pro-inflammatory cargo (such as miRNAs or cytokines) are non-negligible. It is also not known if CAR-EVs may elicit an innate or adaptive immune response, particularly in patients with prior exposure to viral vectors or genetically modified cells. Furthermore, while the use of immortalized cell lines offers a potential solution for scalable and consistent CAR-EV production, this approach has also raised concerns regarding tumorigenicity, genomic instability, and regulatory acceptability, particularly for repeated or high-dose applications. Consequently, there is a pressing need for long-term safety studies and repeat-dosing evaluations to fully understand the risks associated with chronic exposure to engineered EVs derived from such sources.

### 9.4. Regulatory Uncertainty

The regulatory landscape for exosome-based therapeutics, including CAR-EVs, is still evolving. Current key challenges in the field include:

- Classification ambiguity: EVs may fall under different regulatory categories (e.g., biologics, gene therapies, drug-delivery systems) depending on their source, modification method, and intended use.
- Lack of defined regulatory guidance: Unlike cell or gene therapies, there is no dedicated regulatory pathway for EVs. This creates uncertainty in terms of CMC (Chemistry, Manufacturing, and Controls) expectations, nonclinical study design, and clinical trial endpoints. At the symposium “Challenges and Regulations of Extracellular Vesicles in Clinical Development” held on 11 March 2024, a panel comprising experts from academia, industry, and regulatory bodies discussed the evolving regulatory landscape surrounding EV-based therapies. One of the key points raised was the concern over potential overregulation, with some panelists citing Japan as an example where regulatory authorities are reportedly considering applying the same stringent safety standards used for cell-based therapies to EV-based products. While such measures reflect a cautious approach to safety, they also risk delaying innovation and clinical translation. The panel emphasized the urgent need for clearer regulatory guidance, including dedicated frameworks tailored to the unique characteristics of EVs to facilitate their development while ensuring patient safety.
- Biosafety and gene modification concerns: The use of genetically modified parent cells (e.g., lentiviral or CRISPR-edited) raises additional scrutiny from regulators, particularly in jurisdictions where gene therapy regulations are stringent.
- Documentation and traceability: Ensuring full traceability of source materials, including donor cells (if allogeneic), reagents, and exogenous components, is essential for GMP compliance but can be difficult in decentralized or academic manufacturing settings.

## 10. Conclusions

In this review, we explored the multifaceted landscape of CAR-engineered extracellular vesicles, highlighting their therapeutic potential in cancer treatment. As next-generation biologics, CAR-EVs offer distinct advantages, including precision targeting, reduced immunotoxicity, and the potential for off-the-shelf availability. To fully realize their clinical and commercial potential, it will be crucial to foster coordinated efforts among academic researchers, biotechnology developers, and regulatory bodies. These collaborations will be instrumental in refining manufacturing processes, ensuring safety, and establishing scalable, cost-effective solutions that can support the broader implementation of CAR-EV technologies in oncology.

## Figures and Tables

**Table 1:** Overview of FDA-approved CAR-T cell therapies as of October 2025, listing their generic and brand names, approved indications, approval dates, pivotal clinical trials, accelerated approval status, and U.S. wholesale acquisition costs (WAC). Indications include various relapsed/refractory hematologic malignancies such as B cell acute lymphoblastic leukemia (ALL), diffuse large B cell (DLBCL), follicular lymphoma (FL), mantle cell lymphoma (MCL), and multiple myeloma (MM). Some therapies have multiple indications with distinct trial data and pricing [37].

Name	Brand Name	Approved Indication	Approval Date	Accelerated Approval	Pivotal Trial	List Price (WAC, Mar 2023, US$)
Tisagenlecleucel (tisa-cel)	Kymriah	R/R pediatric and young adult (<25) B cell ALL	30 August 2017	No	ELIANA	$543,828
		R/R adult DLBCL, HGBL, transformed DLBCL	1 May 2018	No	JULIET	$427,048
		R/R FL	27 May 2022	No	ELARA	-
Axicabtagene ciloleucel (axi-cel)	Yescarta	R/R DLBCL	18 October 2017	No	ZUMA-1	$424,000
		R/R FL	2 April 2021	Yes	ZUMA-5	$424,000
Lisocabtagene maraleucel (liso-cel)	Breyanzi	R/R DLBCL, HGBL, transformed DLBCL	5 February 2021	Yes	TRANSCEND-NHL-001	$447,227
Brexucabtagene autoleucel	Tecartus	R/R MCL	24 July 2020	No	ZUMA-2	$424,000
		Adult ALL	1 October 2021	Yes	ZUMA-3	$424,000
Idecabtagene vicleucel	Abecma	R/R MM	26 March 2021	Yes	KarMMa	$457,255
Ciltacabtagene autoleucel	Carvykti	R/R MM	28 February 2022	Yes	CARTITUDE	$465,000
Obecabtagene autoleucel	Aucatzyl	R/R ALL	8 November 2024	No	FELIX	-

**Table 2:** Main differences between CAR-immune cell and CAR-extracellular vesicle therapies.

Feature	CAR-Immune Cells	CAR-Extracellular Vesicles (CAR-EVs)
Mechanism of Action	Live immune cells engineered to recognize and kill cancer cells via CARs	Cell-derived extracellular vesicles displaying CARs or containing CAR-related cargo
Tumor Targeting	High specificity to target antigen	High specificity (inherited from parental CAR cell)
Immune System Activation	Strong activation; cytokine release, T cell expansion	Reduced immune activation; lower systemic cytokine response
Toxicity Risk	High; cytokine release syndrome, neurotoxicity, GVHD especially in the case of T cells	Low; non-proliferative and cell-free
Tumor Penetration	Limited in solid tumors due to size and TME	Facilitate due to small size and ability to diffuse into solid tumor tissues
Manufacturing Complexity	Complex, patient-specific (autologous), expensive	Potentially scalable and “off-the-shelf”
Persistence in Body	Long-term persistence (can be beneficial or harmful)	Short-lived; may require repeat dosing
Cargo Delivery	Primarily cell-mediated killing	Can be engineered to deliver miRNAs, mRNA, cytokines, drugs or other bioactive molecules
Storage and Stability	Requires cryopreservation and careful handling	More stable, easier to store and transport
Regulatory Status	Approved therapies exist; well-established frameworks	Experimental; currently lacks clear regulatory classification
Clinical Evidence	>800 trials; several approved products	Mostly preclinical; few early-stage trials
Challenges	High cost, toxicities, limited efficacy in solid tumors	Low in vivo persistence, production variability, regulatory path in development

## Data Availability

No new data were created or analyzed in this study. Data sharing is not applicable to this article.

## Abbreviations

The following abbreviations are used in this manuscript:

ALL	Acute lymphoblastic leukemia
AICD	Activation-induced cell death
BBB	Blood–brain barrier
CAR	Chimeric antigen receptor
CAR-M-EV	Macrophage-derived CAR-EVs
CEA	Carcinoembryonic antigen
CNS	Central nervous system
CRES	CAR-T-related encephalopathy syndrome
CRS	Cytokine release syndrome
CTL	Cytotoxic T lymphocytes
DLBCL	Diffuse large B cell lymphoma
EGFR	Epidermal growth factor receptor
EV	Extracellular Vesicles
FasL	Fas ligand
FL	Follicular lymphoma
GBM	Glioblastoma
GGBB	Geranylgeranyl pyrophosphate
GMP	Good Manufacturing Practice
GvHD	Graft-versus-host disease
HER2	Human epidermal growth factor receptor 2
HLA-E	Human leukocyte antigen E
ICANS	Immune effector cell-associated neurotoxicity syndrome
IFN-γ	Interferon gamma
IL	Interleukin
IS	Immune synapse
IEX	Ion exchange chromatography
KRAS	Kirsten rat sarcoma viral oncogene homolog
LAG-3	Lymphocyte-activation gene 3
LDLR	Low-density lipoprotein receptor
MCL	Mantle cell lymphoma
MDS	Myelodysplastic syndromes
MDSC	Myeloid-derived suppressor cell
MHC	Major histocompatibility complex
MICA	MHC class I chain-related protein A
MICB	MHC class I chain-related protein B
MM	Multiple myeloma
MMC	Multimodal chromatography
MSC	Mesenchymal stem cell
NK	Natural killer
PD-1	Programmed cell death protein 1
PDAC	Pancreatic ductal adenocarcinoma
PD-L1	Programmed death-ligand 1
scFvs	Single-chain variable fragments
SEC	Size-exclusion chromatography
siRNA	Small interfering RNA
TAA	Targeting tumor-associated antigens
TAMs	Tumor-associated macrophages
TCR	T cell receptor
TFF	Tangential flow filtration
TGF β	Transforming growth factor-β
TIM-3	T cell immunoglobulin and mucin-domain containing-3
TME	Tumor microenvironment
TNF-α	Tumor necrosis factor alpha
Tregs	Regulatory T cells
TRUCKs	T cells redirected for universal cytokine killing
T SCM	Stem cell memory T cells
